# The Impact of Two Different Insulin Dose Calculation Methods on Postprandial Glycemia After a Mixed Meal in Children with Type 1 Diabetes: A Randomized Study

**DOI:** 10.3390/nu17203287

**Published:** 2025-10-20

**Authors:** Magdalena Dymińska, Emilia Kowalczyk-Korcz, Katarzyna Piechowiak, Agnieszka Szypowska

**Affiliations:** Department of Pediatric Diabetology, Józef Polikarp Brudziński Public Pediatric Clinical Hospital, University Clinical Center of the Medical University of Warsaw, 02-091 Warsaw, Poland; magdalena.dyminska@uckwum.pl (M.D.); emilia.kowalczyk@uckwum.pl (E.K.-K.); katarzyna.piechowiak@uckwum.pl (K.P.)

**Keywords:** type 1 diabetes, postprandial glycemia, mixed meal, extended bolus, dual wave bolus

## Abstract

**Background/Objectives**: Optimal postprandial glycemic control is crucial to maintain time in range (TIR:3.9–10.0 mmol/L, 70–180 mg/dL) and time in tight range (TITR:3.9–7.8 mmol/L, 70–140 mg/dL), both important to reduce microvascular complications in type 1 diabetes mellitus (T1DM). However, insulin dosing based on carbohydrate counting fails to compensate for delayed hyperglycemia from protein and fat. This study evaluated two advanced insulin dosing algorithms designed to improve postprandial control in adolescents with T1DM. **Methods**: In this randomized, prospective, double-blind, crossover trial, 58 adolescents with T1DM (median age 15.5 years) were enrolled, all using continuous subcutaneous insulin infusion and a continuous glucose monitoring system in non-automated mode. For two consecutive days, participants consumed standardized mixed meals for breakfast (50 g of carbohydrates, 200 kcal from protein and fat) and received an extended bolus delivered for four hours, based on the Pankowska Equation (PE, i.e., Fat-Protein Units × Insulin-to-Carbohydrate Ratio (ICR)) and the Sieradzki Equation (SE, i.e., 30% × Carbohydrate Units × ICR). Postprandial glucose was monitored for five hours using a glucometer and Continuous Glucose Monitoring (CGM). The primary outcome was the capillary blood glucose level at predefined time points. The secondary outcomes were the frequency of hypoglycemia and glycemic variability parameters. **Results**: Both methods kept postprandial glucose within the recommended TIR. The SE method provided longer TITR (82.51% vs. 70.49%, *p* = 0.6281) and fewer hypoglycemic episodes at 180 and 300 min. Glucose levels at 60 min, were higher after PE (136 ± 35.2 mg/dL vs. 124 ± 32.2 mg/dL, *p* = 0.016). **Conclusions**: Both algorithms provided effective postprandial control after a mixed meal, but SE achieved a longer TITR and fewer late hypoglycemic events.

## 1. Introduction

The primary objective of therapeutic management in type 1 diabetes mellitus (T1DM) is to achieve optimal glycemic control to prevent both acute and long-term complications. Postprandial glycemia plays a crucial role in overall glycemic control and variability, significantly impacting long-term metabolic outcomes [1,2]. A key therapeutic goal is to minimize glycemic variability, while maximizing time in range (TIR: 3.9–10.0 mmol/L, 70–180 mg/dL) and time in tight range (TITR: 3.9–7.8 mmol/L, 70–140 mg/dL) especially for people with advanced technology support, such as continuous glucose monitoring system (CGM) and automated insulin delivery (AID) [3]. Evidence and guidelines suggest that higher TITR and TIR quartiles are associated with a lower prevalence of microvascular complications, including diabetic retinopathy, nephropathy, and cerebrovascular events. Each 10% increase in TITR correlates with a reduced risk of microvascular complications (OR 0.762; 95% CI 0.679–0.855; *p* < 0.001) [4].

High-protein and high-fat meals significantly modify postprandial glycemia in individuals with T1DM, leading to delayed and sustained glycemic excursion [5,6,7,8,9,10]. Evidence from multiple studies indicates that macronutrients increase insulin requirements, and that extended or additional insulin dosing strategies are more effective in achieving postprandial glycemic control, particularly for mixed meals containing carbohydrates, protein and fat [10,11,12]. Postprandial glycemic control significantly impacts TIR and TITR, and is particularly challenging in individuals with T1DM who use non-automated insulin pumps, especially after consuming mixed meals with high protein and fat content.

Traditional insulin dosing algorithms focus primarily on carbohydrate counting, often failing to adequately compensate for delayed hyperglycemia caused by prolonged digestion and metabolic conversion of protein and fat [6]. A recent meta-analysis demonstrated the benefits of incorporating additional insulin for fat and protein counting, with recommended supplementary doses ranging from 24% to 75% of the insulin-to-carbohydrate ratio (ICR), administered as a combination bolus, with a minimum upfront dose of 60% of the ICR. The analyzed studies had wide variations in fat (2–79 g) and protein (10–60 g) content in meals, which could have influenced insulin requirements [13]. Lopez et al. evaluated five different bolus combinations and demonstrated that an additional insulin at an insulin-to-carbohydrate ratio of up to 70% is required in an extended bolus for high-fat and high-protein meals to prevent delayed hyperglycemia [14].

The daily diet of adolescents with T1DM should consist of balanced mixed meals that provide approximately 45% of total caloric intake from carbohydrates [15]. However, studies determining the most effective insulin dosing equations for fat and protein counting in balanced mixed meals to reduce glycemic variability in everyday dietary management are limited.

Although the ISPAD guidelines recommend extended boluses for meals containing fat and protein, there is no clear consensus on the optimal duration of such boluses [15].

In Poland, the Warsaw Insulin Dosing System, also known as the Pańkowska Equation, accounts for both carbohydrate content and caloric intake from protein and fat. According to this method, the extended bolus insulin dose required for 100 kcal derived from protein and fat (one fat-protein unit, FPU) is equivalent to that needed for 10 g of carbohydrates, provided the fat-protein content exceeds 100 kcal [16].

Some previous studies have shown that the use of the Pankowska equation results in an improved postprandial glycemic profile, but is associated with a significantly higher number of hypoglycemic events [17,18,19]. However, others have not confirmed this effect [20,21].

Moreover, this method requires calculating the caloric content of each meal, which is complex and can be difficult for adolescents with T1DM to apply in daily practice.

In search of a less complicated method of determining the insulin dose for an extended bolus to cover fat and protein, we compared the commonly used Pańkowska equation with an alternative approach suggested by Sieradzki (the Sieradzki equation) [22]. The alternative method involves administering an additional extended bolus dose for fat and protein, equivalent to 30% of the mealtime insulin dose, calculated based solely on carbohydrate content.

The aim of the study is to determine the optimal bolus split for maintaining postprandial glycemia with a balanced mixed meal in adolescents with type 1 diabetes using non-automated insulin pumps and CGM.

## 2. Materials and Methods

The study was designed as a prospective, randomized, double-blind crossover trial with a 1:1 allocation. It was conducted from October 2019 to March 2024 in the Department of Pediatric Diabetology and Pediatrics at the University Clinical Centre of the Medical University of Warsaw, Poland. The trial was approved by the Ethics Committee of the Medical University of Warsaw, Poland (approval number KB/73/2019). The protocol was registered at ClinicalTrials.gov (https://clinicaltrials.gov/study/NCT04124302, accessed on 23 August 2019; identifying number NCT04124302) before including the first participant. De-identified individual participant data will be available from the corresponding author upon reasonable request, starting six months after publication and continuing for up to five years. Neither participants nor members of the public were involved in the design, conduct, or reporting of this trial. Informed consent was obtained from all participants and their caregivers prior to the study enrollment. The study included children and adolescents between the ages of 10 and 18 who had T1DM for at least one year and had been treated with continuous subcutaneous insulin infusion (CSII) and one type of insulin for at least three months. The exclusion criteria were: coeliac disease, diabetes-related complications (e.g., nephropathy), use of faster aspart insulin, withdrawal of consent, and any disease that the investigator deemed to affect the trial. The eligibility and exclusion criteria for participants did not change during the study. All participants were hospitalized before and during the study. Those who met the eligibility criteria were hospitalized for approximately one week prior to the study to review their daily glycemic profile. If necessary, adjustments were made to the insulin regimen to optimize the fasting blood glucose (FBG) level and determine the individualized ICR. For two consecutive days, the study participants had to maintain fasting glycemia within the range of 70 to 130 mg/dL (3.9–7.2 mmol/L). They were considered to have achieved normoglycemia if their blood glucose level was <140 mg/dL (<7.8 mmol/L) two hours after breakfast. This confirmed that the ICR and daily insulin dose were correctly adapted. Only then was the participant included in the study. One day before the beginning of the study, each participant was equipped with a continuous glucose monitoring (CGM) system, Enlite™ sensor (Medtronic MiniMed, Northridge, CA, USA) with a MiniLink™ Transmitter (Medtronic MiniMed, Northridge, CA, USA), and a MiniMed^TM^ VEO™ insulin pump (Medtronic MiniMed, Northridge, CA, USA). The participants had to have fasting glycemia between 70 and 130 mg/dL (3.9–7.2 mmol/L) with no glycemic fluctuations or correction boluses for at least three hours before the test meal consumption on both days of the study.

The test meal consisted of white toast bread (96 g), Maasdam cheese (44 g) and tomato ketchup (8 g). It contained 50% of carbohydrates, 30% of fat and 20% of protein, equaling 50 g of carbohydrates, 200 kcal from fat and protein. All participants received the test meal on two consecutive mornings. They were randomized into two groups using a block randomization process with blocks of four. A computer program (V.3.1.4, StatsDirect, Cheshire, UK) generated the randomization list which was kept in envelopes. The investigator was provided with randomly generated treatment allocations in sealed, opaque envelopes. Upon the participant’s inclusion in the trial, the corresponding envelope was opened and the assigned treatment was administered. All participants and investigators were blinded. The insulin boluses were administered by nurses not involved in the study. After the bolus administration, the pump screen was covered with non-transparent tape to prevent participants from viewing the sensor glucose readings on the pump display, thereby preventing any influence on their behavior during the observation period. All participants were randomly assigned to one of two groups, depending on the sequence of boluses they received during the two-day intervention: the PE-Group or the SE-Group. On the first day of the trial, the PE Group received an insulin bolus 15 min before breakfast, calculated using the Pańkowska equation (PE). The insulin dose consisted of:

- A standard bolus covering carbohydrates, calculated as Carbohydrate Units × ICR
- An extended bolus covering fat and protein, calculated as Fat-Protein Units × ICR, delivered for four hours.

One Fat-Protein Unit was defined as 100 kcal derived from fat and protein in a meal, while one Carbohydrate Unit was defined as 10 g of carbohydrates in a meal. The ICR represents the individual insulin-to-carbohydrate ratio.

On the first study day, the SE group received insulin boluses calculated using the Sieradzki equation (SE):

- A standard bolus covering carbohydrates, calculated as Carbohydrate Units × ICR;
- An extended bolus covering fat and protein, calculated as (30% × CU × ICR), delivered for 4 h.

On the second day of the study, the participants in PE Group received insulin boluses calculated using the SE algorithm, while the participants in the SE Group received insulin boluses calculated using the PE algorithm. All boluses were administered 15 min before the meal.

The present study chose a 4 h observation period to standardize insulin delivery, consistent with the Pańkowska protocol, where the duration of the extended bolus corresponds to the calculated FPU plus additional two hours for all participants [20].

**Figure 1:**
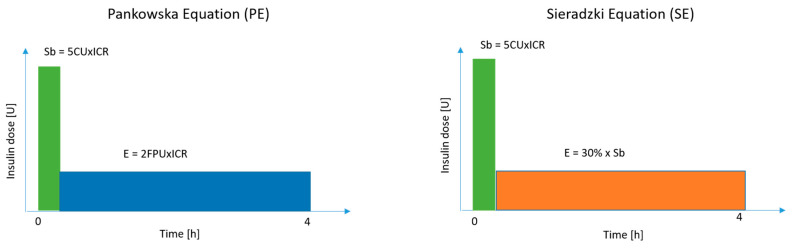
Graphical representation of boluses based on the Pankowska and Sieradzki Equations. Sb—standard bolus; E—extended bolus; CU—carbohydrate units; FPU—fat-protein units; ICR—insulin–carbohydrate ratio; h—hours; U—units.

**Figure 2:**
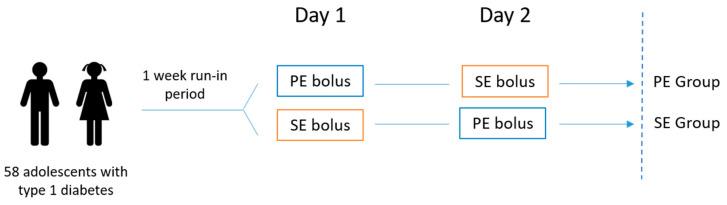
Graphical representation of the study intervention. PE bolus—bolus based on the Pankowska Equation algorithm; SE bolus—bolus based on the Sieradzki Equation algorithm.

The observation period was scheduled for five hours after bolus administration. Figure 1 and Figure 2 provide graphical representations of the bolus administration and the study intervention.

In addition to CGM measurements, participants were asked to test their capillary blood glucose concentrations (BG) at baseline and every 30 min during the five-hour observation using a Contour^TM^ Plus glucometer (Ascensia Diabetes Care, Switzerland), under the supervision of research staff. According to the ISPAD recommendation, hypoglycemia was defined as a glucose level below 70 mg/dL (<3.9 mmol/L), and clinically important hypoglycemia was defined as a glucose level below 54 mg/dL (<3.0 mmol/L). Treatment for hypoglycemia during observation involved administering 0.3 g/kg of glucose orally, up to a maximum of 15 g, which corresponds to the amount of carbohydrates recommended by ISPAD [23].

The primary outcome was postprandial glycemia (PPG) at 60, 120, 180, 210, 240, 270, and 300 min, based on glucometer data.

The secondary outcomes were as follows: hypoglycemic episodes based on glucometer data, hypoglycemic episodes based on CGM data, mean amplitude of glycemic excursion (MAGE), glycemic rise (GR), time in postprandial glucose range (TIR) between 70–180 mg/dL (3.9–10.0 mmol/L, 70–180 mg/dL), time in tight range (TITR) between 70–140 mg/dL (3.9–7.8 mmol/L), time spent in hypoglycemia under 70 mg/dL (<3.9 mmol/L), time spent in hypoglycemia under 54 mg/dL (<3.0 mmol/L), time to peak (TTP), time to nadir glucose (TNG), area under the curve (AUC) using CGM data.

Hypoglycemic episodes were categorized into two groups: time below range (TBR) (1), defined as events with nadir glucose concentration <70 mg/dL (<3.9 mmol/L) but ≥54 mg/dL (≥3.0 mmol/L); and TBR (2), defined as events with glucose levels <54 mg/dL (<3.0 mmol/L). Each category was analyzed independently. For CGM data, an episode of hypoglycemia was defined as a sequence of consecutive readings below the specified threshold, and was considered a single event until the glucose value returned to the target range. Importantly, only episodes lasting at least 15 min were classified as hypoglycemia based on CGM. In contrast, each individual blood glucose measurement obtained by glucometer was considered a separate episode. We considered it one episode of hypoglycemia until the subsequent controlled measurements showed that glucose values had returned to the target range [24,25].

### Statistical Analysis

To detect a 30 mg/dL difference in peak postprandial glucose concentrations at 60, 120, 180, 210, 240, 270, and 300 min (the primary endpoint), a total of 58 participants were required, assuming a standard deviation of 80 mg/dL. To account for an anticipated 20% dropout rate, the target sample size was increased to 72 participants. Sample size calculations were performed using StatsDirect software (V.3.1.4, StatsDirect, Chesire, UK). Analyses were carried out on a per-protocol basis, including all participants who completed the study. The study group was described with appropriate descriptive statistics. The normality of the distribution was verified using Shapiro–Wilk test. Student’s *t*-test for independent samples was used to assess statistical significance between the groups. For non-parametric data, the Mann–Whitney U test or Fisher’s exact test was used, as appropriate. Odds ratios (ORs) with 95% confidence intervals (CIs) were calculated. Quantitative variables were presented as arithmetic means with standard deviations (SDs) for normally distributed data, or as medians with interquartile ranges (IQRs) for non-normally distributed data. The area under the curve (AUC) was calculated using the trapezoidal method, based on continuous glucose monitoring (CGM) data collected over a five-hour period following each test meal. The glucose rise (GR) was defined as the difference between the peak postprandial glucose value and the baseline (pre-meal) glucose concentration, calculated over the 0–300 min postprandial period based on CGM data. Time to peak (TTP) was defined as the interval from the start of the meal (time 0) to the time at which the highest postprandial glucose value was recorded.

Time to nadir glucose (TNG) was defined as the interval from the start of the meal until the lowest postprandial glucose value was observed during the analysis period.

A *p*-value of < 0.05 was considered statistically significant. Statistical analyses were conducted using GraphPad Prism version 10.5.0 (GraphPad Software, La Jolla, CA, USA).

## 3. Results

**Figure 3:**
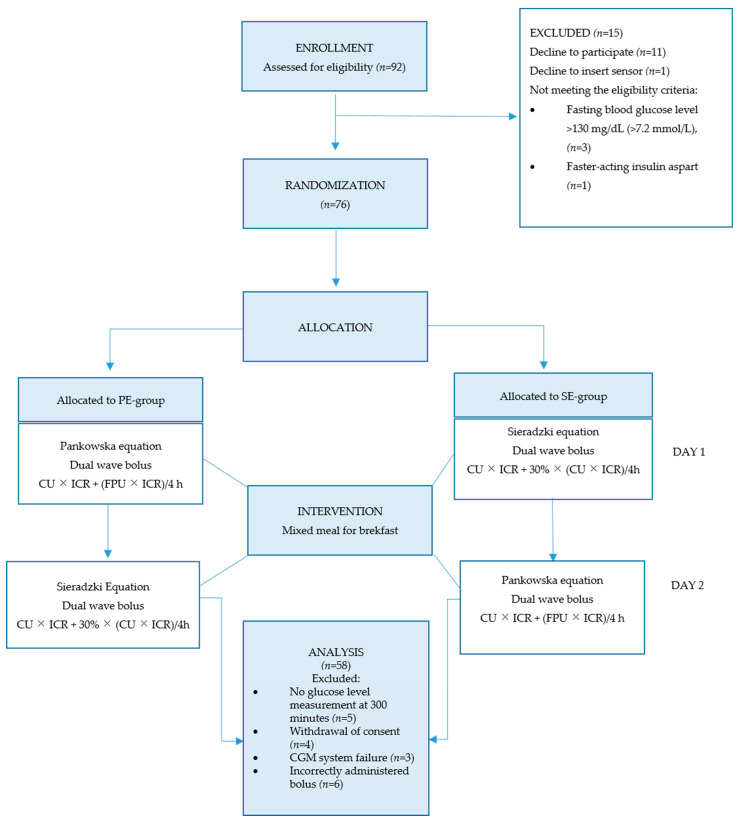
Study flow diagram.

Figure 3 presents the study flow diagram.

Participants with missing outcome data were excluded from the respective analyses (complete case analysis). A total of 76 individuals were randomized, but 18 were excluded from the study due to: a lack of glucose level measurement at 300 min (*n* = 5); CGM system failure, i.e., sensor detachment; interruption of data transmission resulting in more than 50% missing readings; large discrepancies in results from the glucometer and CGM system (*n* = 3); incorrectly administered bolus (*n* = 6); and withdrawal of consent (*n* = 4). In total, 58 adolescents (76.3%) with a median age of 15.51 and balanced gender distribution completed the study. Twenty-seven participants were assigned to the PE-Group and 31 were assigned to the SE-Group. The characteristics of the participants are presented in Table 1. No adverse events occurred during the study.

A statistically significant difference was observed only at 60 min, with higher values recorded in the PE-Group (136 ± 35.2 mg/dL) than in the SE-Group (124 ± 32.2 mg/dL; *p* = 0.0161). There were no significant differences in baseline glucose values or at 30, 90, 120, 180, 210, 240, 270, or 300 min based on capillary blood glucose measurements (*p* > 0.05).

**Figure 4:**
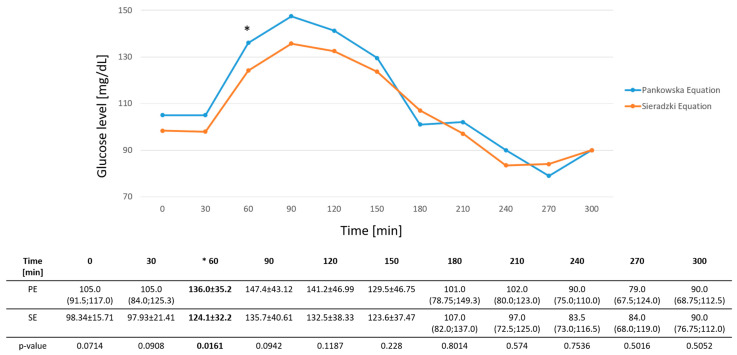
Glucose level after different types of boluses measured by glucometer. PE—Pankowska Equation; SE—Sieradzki Equation; *—indicates a statistically significant difference between the PE and SE groups (*p* < 0.05).

**Figure 5:**
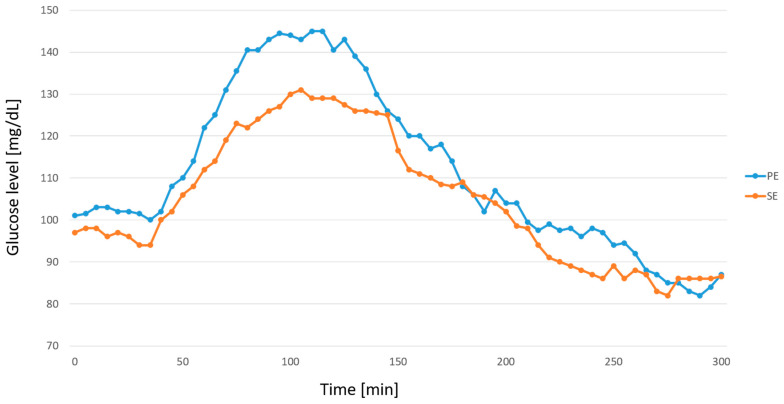
Glucose levels after boluses based on the Pankowska Equation (PE) and the Sieradzki Equation (SE)—values from CGM.

Both groups achieved excellent metabolic control, maintaining glucose concentrations within the recommended target range (TIR) of 70–180 mg/dL. The median TIR was 92.62% (79.26; 100.0) and 96.72% (81.56; 100.0) after PE and SE bolus administration, respectively. After the PE bolus, glucose levels at 90 and 120 min were slightly above the TITR threshold compared to the SE bolus, with values of 147.4 ± 43.12 mg/dL vs. 135.7 ± 40.61 mg/dL (*p* = 0.0942) and 141.2 ± 46.99 vs. 132.5 ± 38.33 mg/dL (*p* = 0.1187), respectively. Figure 4 presents glucose levels measured by glucometer following both boluses. Figure 5 shows corresponding data obtained from CGM.

**Figure 6:**
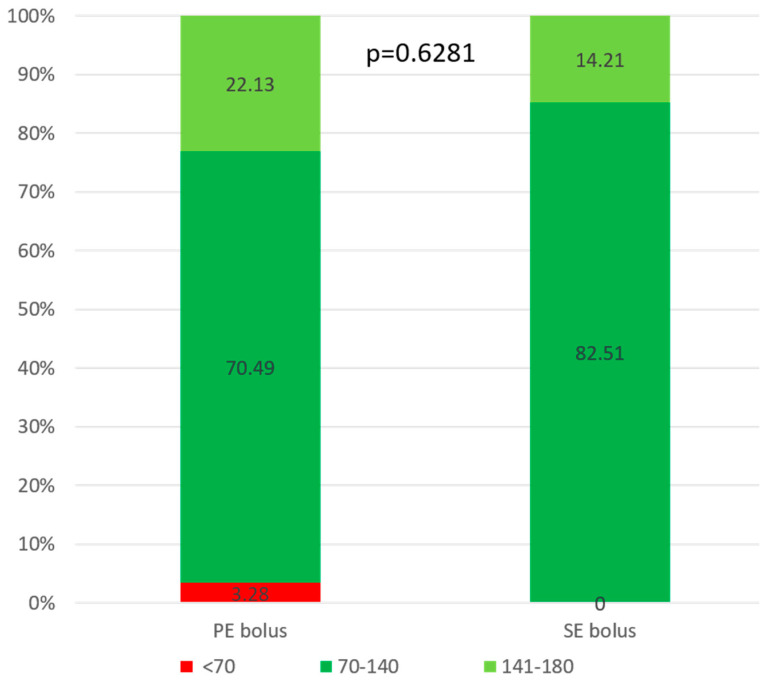
Comparison between groups: mean time in tight range (70–140 mg/dL), time in range (70–180 mg/dL), above (>180 mg/dL) and below range (<70 mg/dL).

During the observation period, the recommended TITR was achieved in both groups, 70. 49% (49.59; 87.30) and 82.51% (62.3; 96.72) after the PE and SE boluses, respectively (*p* = 0.6281). However, the group that received the SE bolus achieved a statistically longer TITR (*p* = 0.0139). Figure 6 shows the comparison of mean time in range between the groups.

**Figure 7:**
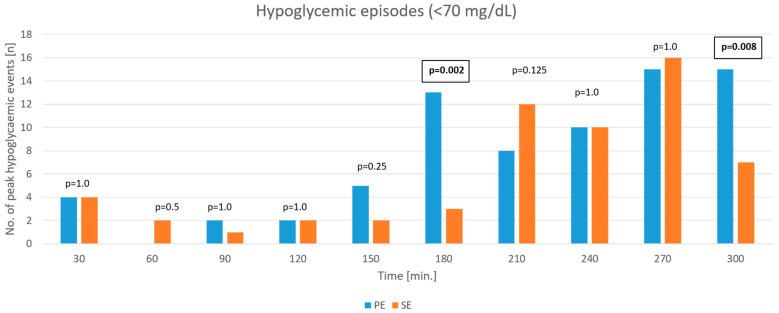
Comparison of hypoglycemic events (<70 mg/dL) at postprandial time points based on blood glucose concentrations, after bolus administration, calculated using the Pankowska Equation (PE) and the Sieradzki Equation (SE).

The overall number of the study participants with hypoglycemia <70 mg/dL (*p* = 0.2673) and <54 mg/dL (*p* = 0.1824) after both types of bolus, based on BG and CGM measurements <70 mg/dL (*p* = 0.998 and *p* = 0.50, respectively) did not differ significantly between the PE and SE groups. The total time spent below target range based on CGM, after both types of bolus, was also comparable (<70 mg/dL (*p* = 0.8174), <54 mg/dL (*p* = 0.50)). However, time-point analysis revealed statistically significant differences at 180 and 300 min. The number of hypoglycemic episodes was significantly higher at both time points after the PE bolus compared to the SE bolus: 13 vs. 3 events (*p* = 0.002) and 15 vs. 7 events (*p* = 0.008) at 180 and 300 min, respectively. Figure 7 shows hypoglycemic events at specific time points. We did not find any significant differences in <54 mg/dL hypoglycemic episodes at any time point (*p* > 0.05).

**Figure 8:**
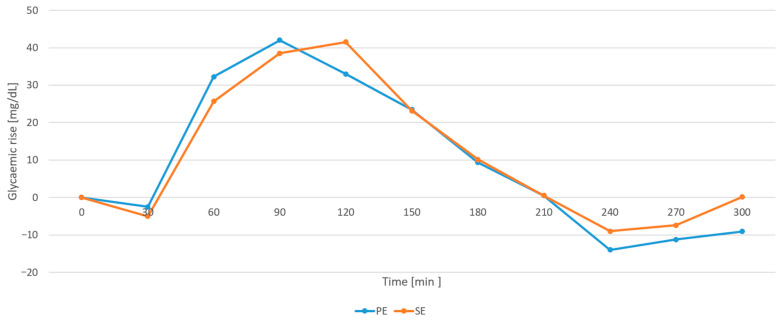
Change in glycemia (glycemic rise, GR) from the baseline value after the bolus based on the Pankowska Equation (PE) and the Sieradzki Equation (SE).

There were no significant differences between the groups in terms of AUC (*p* = 0.623), TTP (*p* = 0.6161), TNG (*p* = 0.8795), MAGE (*p* = 0.1727) or GR (p>0.05). The results are presented in Table 2. The median GR values at each time point are shown in Figure 8.

## 4. Discussion

During the observation period, both bolus administration strategies enabled the study participants to maintain glucose levels within the recommended target range (TIR) of 70–180 mg/dL during the five-hour observation period after breakfast. However, the SE method was associated with significantly better outcomes. The median glucose target range of 70–140 mg/dL (TITR) was 70.49% (49.59; 87.30) following PE administration and 82.51% (62.30; 96.72) following SE administration. The difference between the groups was statistically significant (*p* = 0.0139), indicating that the SE method provided a more sustained period of glycemic control within the recommended range. These findings suggest that the SE strategy may be advantageous for optimizing postprandial glucose management after a mixed meal for breakfast.

The study demonstrated that, at most time points, glucose concentrations were comparable between the groups, except for a statistically significant difference at 60 min, when higher glucose levels were recorded in the PE group than in the SE group. Although baseline fasting glucose levels differed slightly between the groups (*p* = 0.07), all participants followed an identical run-in protocol, and baseline values remained within the recommended target range. Although baseline fasting glucose levels tended to differ between the study arms, this minor imbalance may have contributed to the early postprandial difference observed at 60 min. However, it is unlikely to have substantially influenced the overall postprandial outcomes.

The main finding of this study was the absence of statistically significant differences in postprandial glycemia following the administration of an extended bolus for a 400 kcal balanced breakfast (with 50% of the calories derived from carbohydrates and 50% from protein and fat), regardless of the insulin dosing method, whether calculated according to the Pankowska Equation (PE) or by adding 30% of the carbohydrate-based insulin dose, as per the Sieradzki Equation (SE).

Clinical experience and patient reports suggest that breakfast typically requires a higher insulin dose compared to other meals. Despite pre-meal insulin administration, children often experience significant postprandial glucose excursions following breakfast. This makes it particularly difficult to maintain blood glucose levels within the target range of 70–140 mg/dL.

In our study, the mean glucose values at two hours after breakfast met the therapeutic target of 140 mg/dL (7.8 mmol/L) in the SE group, as recommended by the Polish Diabetes Association (PTD) [26]. Other authors have indicated that administering an additional insulin dose with protein- and fat-containing meals helps prevent postprandial hyperglycemia. Paterson et al. noted that high-protein meals require 30% more insulin to prevent delayed postprandial hyperglycemia [27]. Smith et al. demonstrated that an additional 40% of the insulin dose for a high-fat, high-protein breakfast improves postprandial glycemic excursions in children and young adults with T1DM using pump therapy [28].

Recent evidence shows that higher TIR and TITR values are associated with a lower risk of both micro- and macro-vascular complications. De Meulemeester et al. found a 10% increase in TITR to be linked with a significant reduction in the prevalence of microvascular complications, such as retinopathy, neuropathy, nephropathy, and stroke [4]. Similar results were demonstrated by Beck et al., who confirmed that a lower TIR value significantly increases the risk of developing retinopathy and microalbuminuria [29]. Increasing TIR by just 5% has been shown to have clinical effects, including reductions in the risk of microvascular complications and improved quality of life [24].

Both dosing methods resulted in a comparable overall number of hypoglycemic episodes, though statistically significant differences were noted at specific time points. In both groups, a significant increase in hypoglycemic events was observed between three and five hours postprandially. The lower incidence of hypoglycemia after the SE bolus at 180 and 300 min postprandially suggests that the SE bolus provides more balanced insulin delivery, reducing the risk of late postprandial hypoglycemia. No severe hypoglycemic episodes were recorded.

Automated insulin delivery systems (AID) are now strongly recommended for young people with diabetes. To avoid hypoglycemia, insulin dosing should be tailored to each patient’s needs and preferences. Modern AID systems reduce the risk of hypoglycemia by suspending basal insulin infusion and delivering automated correction boluses. They also adjust basal rates dynamically, thereby reducing late postprandial hyperglycemia [30]. Although AID systems are increasingly available, their current algorithms do not account for mixed-meal composition, limiting their ability to accurately predict the postprandial glycemic response [31,32]. Future studies should investigate whether current AID algorithms adequately account for the metabolic effects of dietary fat and protein, as well as whether further insulin adjustments are necessary under hybrid closed-loop conditions.

### Strengths and Limitations

This study has several notable strengths. First, this randomized, double-blind, crossover trial was conducted under controlled inpatient conditions, ensuring high internal validity and a standardized assessment of postprandial glycemia. By combining CGM and BG monitoring, it provided a comprehensive evaluation of glycemic outcomes. The study addresses the clinically relevant question of how to optimize insulin dosing beyond carbohydrate counting by incorporating fat and protein content.

Several limitations are noteworthy. First, the study was performed using only one type of open-loop insulin pump, the MiniMed^TM^ VEO™ (Medtronic MiniMed, Northridge, CA, USA). This may limit the generalizability of our findings.

The observation period was limited to a single meal, which does not capture the variability of glycemic responses to different meal compositions. Another limitation is that the meal was eaten only at one specific time. A third arm of the study is needed to evaluate postprandial glucose control after the same meal without the extended bolus. The observation period was limited to five hours, so late glucose changes could not be detected. Furthermore, while the controlled hospital setting ensured internal validity, it may not fully reflect real-life conditions.

## 5. Conclusions

In young people with T1DM using open-loop insulin pump systems, an additional extended bolus equal to 30% of the prandial insulin dose calculated for carbohydrate content, according to the Sieradzki Equation, may represent a simple and practical strategy to improve postprandial glycemic control after mixed breakfast meals. However, this approach should be considered as part of individualized diabetes management only provided that individuals are adequately trained to estimate macronutrient composition. Further studies are needed to confirm its effectiveness for other meal types and in different daily settings.

## Figures and Tables

**Table 1:** Baseline characteristics of the study group.

Characteristic	N	N(%)/Mean ± SD/Median (Q1;Q3)
Sex, *n* (%)	58	
Female		33 (56.9)
Male		25 (43.1)
Age (years) ^1^	58	15.51 (13.21; 16.91)
Duration of disease (years) ^2^	58	8.9 ± 3.76
BMI (kg/m^2^) ^1^	58	20.89 (19.1; 24.23)
BMI Z-score ^2^	58	0.34 ± 0.82
TDD/kg (u/kg) ^2^	58	0.8 ± 0.23
Base/kg (u/kg) ^2^	58	0.32 ± 0.12
Base/TDD (%) ^2^	58	40.36 ± 9.12
HbA1c (%) ^1^	58	8.2 (7.4; 9.3)
Breakfast’s ICR (u) ^2^	58	1.58 ± 0.52
Insuin type, *n* (%)	58	
NovoRapid, Novo Nordisc		23 (39.66)
Liprolog, Eli Lilly		17 (29.31)
Humalog, Eli Lilly		10 (17.24)
Apidra, Sanofi		8 (13.79)

Data are presented as median (Q1; Q3) ^1^ or mean ± SD ^2^, unless stated otherwise. BMI—Body Mass Index; TDD/kg—total daily insulin dose calculated per kg of body mass; Base/kg—basal insulin dose calculated per kg of body mass; Base/TDD—ratio of basal insulin to total daily insulin dose; HbA1c—glycated hemoglobin; ICR—insulin–carbohydrate ratio (defined as the dose of insulin necessary to cover 10 g of carbohydrates); u—unit.

**Table 2:** The comparison between the Pankowska Equation (PE) and the Sieradzki Equation (SE) for the area under the curve (AUC), time to peak (TTP), time to nadir glucose (TNG), and mean amplitude of glycemic excursion (MAGE).

	PE	SE	*p*-Value	Statistically Significant
AUC [mg/dL × min]	5292	5004	0.2117	N
TTP [min]	105 (88.75; 151.3)	115 (90.0; 157.5)	0.6161	
TNG [min]	235 (151.3; 281.3)	227.5 (108.8; 281.3)	0.8795	
MAGE [mg/dL]	112.6 (101.4; 132.1)	107.0 (95.9; 118.5)	0.1727	

Data presented as median (Q1;Q3).

## Data Availability

The data presented in this study are available on request from the corresponding author due to privacy and ethical restrictions.

## Abbreviations

AID—automated insulin delivery; AUC—area under the curve; BG—blood glucose concentrations; CGM—continuous glucose monitoring system; CSII—continuous subcutaneous insulin infusion; CU—Carbohydrate Units; FBG—fasting blood glucose level; GR—glycemic rise; ICR—insulin-to-carbohydrate ratio; MAGE—mean amplitude of glyemic excursion; PE—Pankowska equation; PPG—postprandial glycemia; PTD—Polish Diabetes Association; SE—Sieradzki equation; T1DM—type 1 diabetes mellitus; TITR—time in tight range; TIR—time in range; TNG—Time to nadir glucose; TTP—Time to peak.
